# Incidentally Diagnosed Extraluminal Leiomyosarcoma of Infrarenal Inferior Vena Cava: A Case Report and Literature Review from a Radiologist’s Perspective

**DOI:** 10.15388/Amed.2022.29.2.12

**Published:** 2022-06-29

**Authors:** A Ebinesh, Aanchal Ashta, Satyam, Gaurav Shanker Pradhan, Rohin Sharma, Prince Das

**Affiliations:** Department of Radiodiagnosis, Maulana Azad Medical College and associated hospitals, Jawahar Lal Nehru Marg, New Delhi, India; Department of Radiology, Maulana Azad Medical College and associated hospitals, Jawahar Lal Nehru Marg, New Delhi, India; Department of Radiodiagnosis, Maulana Azad Medical College and associated hospitals, Jawahar Lal Nehru Marg, New Delhi, India; Department of Radiodiagnosis, Maulana Azad Medical College and associated hospitals, Jawahar Lal Nehru Marg, New Delhi, India; Department of Radiodiagnosis, Maulana Azad Medical College and associated hospitals, Jawahar Lal Nehru Marg, New Delhi, India; Department of Radiology, Maulana Azad Medical College and associated hospitals, Jawahar Lal Nehru Marg, New Delhi-110002, India

**Keywords:** Leiomyosarcoma, inferior vena cava, retroperitoneal tumor, imaging features

## Abstract

**Background:**

Vascular leiomyosarcoma is a rare but most common vascular tumor of the inferior vena cava.

**Case presentation:**

We present the case of an incidentally diagnosed extraluminal leiomyosarcoma of the inferior vena cava in a 62 year old patient who presented with abdominal pain following blunt trauma. Ultrasonography showed a lobulated hypoechoic lesion in the upper abdomen. Computed tomography (CT) and magnetic resonance imaging (MRI) showed a circumscribed lobulated near homogeneously enhancing retroperitoneal lesion in anterior relation to the infrarenal inferior vena cava, right paramedian in location with imperceptible vena caval lumen at the site of maximum contact. In positron emission tomography (PET) CT the lesion showed mild fluorodeoxyglucose (FDG) uptake with no distant metastases. CT guided biopsy with immunohistochemical analysis showed leiomyosarcoma. Patient underwent surgical resection with inferior vena cava reconstruction.

**Conclusions:**

Leiomyosarcoma of the inferior vena cava is a rare tumor of vascular origin. Imaging plays an imperative role in the diagnosis and preoperative evaluation. This article also provides a comprehensive literature review of the radiological features of inferior vena caval leiomyosarcoma that would aid in optimal preoperative characterization and evaluation.

## Santrauka.

**Įvadas:**

Kraujagyslių lejomiosarkoma yra retas, tačiau dažniausiai pasitaikantis apatinės tuščiosios venos navikas.

**Atvejo aprašymas:**

62 metų pacientui, kuris kreipėsi į gydymo įstaigą dėl pilvo srities skausmo po neaštriu daiktu sukeltos traumos, buvo atsitiktinai nustatyta neluminalinės infrarenalinės apatinės tuščiosios venos lejomiosarkoma.

Ultragarso tyrimas atskleidė skiltinį hipoechogenišką pažeidimą viršutinėje pilvo srityje. Kompiuterinė tomografija (KT) ir magnetinio rezonanso tomografija (MRT) parodė, kad priešakinėje infrarenalinėje apatinėje tuščiojoje venoje yra apibrėžtas skiltinis, beveik homogeniškai intensyvėjantis retroperitoninis pažeidimas, kuris didžiausio sąlyčio vietoje su tuščiosios venos spindžiu į dešinę nuo centro yra beveik nematomas. Pozitronų emisijos tomografijos (PET) metu pažeidimas parodė nedidelį fluorodeoksigliukozės (FDG) įsisavinimą be jokių tolimų metastazių. Atlikus kompiuterine tomografija paremta biopsiją su imunohistochemine analize, nustatyta lejomiosarkoma. Pacientui buvo atlikta chirurginė rezekcija ir apatinės tuščiosios venos rekonstrukcija.

**Išvados:**

Apatinės tuščiosios venos lejomiosarkoma yra retas kraujagyslių sistemos auglys. Diagnozei ir vertinimui iki operacijos būtina peržiūrėti vaizdinę medžiagą. Šiame straipsnyje taip pat pateikiama išsami literatūros apžvalga apie radiologines apatinės tuščiosios venos lejomiosarkomos savybes, kurios pagerintų optimalų priešoperacinį įvertinimą.

## Introduction

Leiomyosarcoma (LMS) of inferior vena cava is a rare malignant tumor originating from the smooth muscle layer of the tunica media. It was first reported by Paerl et al [1] in 1871. Leiomyosarcomas of vascular origin are rare with an incidence of less than 2% [2]. Occurrence of leiomyosarcoma of the inferior vena cava is exceedingly rare, accounting for 0.5 % of all adult sarcomas [3]. These tumors are asymptomatic or present with nonspecific symptoms such as anorexia, vomiting, loss of weight and abdominal pain and shows female preponderance. At rare instances, the presentation as Budd–Chiari syndrome has also been reported earlier [4,5]. Definitive treatment is surgical excision and prognosis is generally poor [6].

In this report, we present the case of an extraluminal leiomyosarcoma incidentally diagnosed in a 62 year old male patient who presented with abdominal pain following blunt abdominal trauma. Owing to the paucity of literature on radiological features of these tumors, we also present a comprehensive review of the crucial radiological aspects for evaluation.

## Case report

A 62 year old male presented with complaints of pain in abdomen following blunt trauma to abdomen. On examination, he was afebrile with stable vitals. Palpation of the abdomen revealed tenderness in the upper abdomen and a firm palpable mass along the midline of upper abdomen.

Routine ultrasonographic examination revealed a circumscribed hypoechoic lesion in the upper abdomen measuring 6.6 × 6.3 × 5.9 cm (CC × AP × Tr). For further evaluation, patient underwent computed tomography which revealed a circumscribed retroperitoneal soft tissue lesion in right side of midline, measuring 7.3 × 7.5 × 8.1cm (CC × AP × Tr). The lesion showed inhomogeneous contrast uptake on arterial phase and homogeneous enhancement on portal venous and delayed phases with few nonenhancing areas within. It was abutting the inferior vena cava posteriorly with imperceptible lumen at the site of maximum contact. However, proximal and distal segments of the inferior vena cava showed normal contrast opacification. Medially, the lesion was in relation to the abdominal aorta. Second and third part of duodenum and head of pancreas were displaced anterosuperiorly. Magnetic resonance imaging (MRI) showed a homogeneously enhancing circumscribed lesion in the retroperitoneum on right side of midline appearing isointense on T1W, hyperintense on T2W showing restricted diffusion. Few cystic areas were seen within the lesion. On prone imaging, the lumen of inferior vena cava was still imperceptible at the site of maximum contact. No obvious intraluminal extension was seen. Whole body positron emission tomography CT revealed mild FDG uptake with a maximum standard uptake value (SUV_max_) of 4.1. No distant metastases were found.

**Figure 1.**
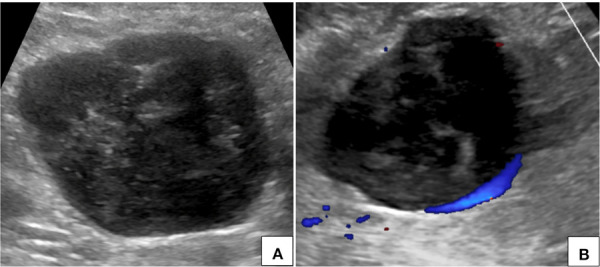
Ultrasonography of abdomen (A) with color Doppler flow imaging (B) shows a heterogeneously hypoechoic circumscribed lesion in the upper abdomen in right paramedian location with no significant internal vascularity.

**Figure 2.**
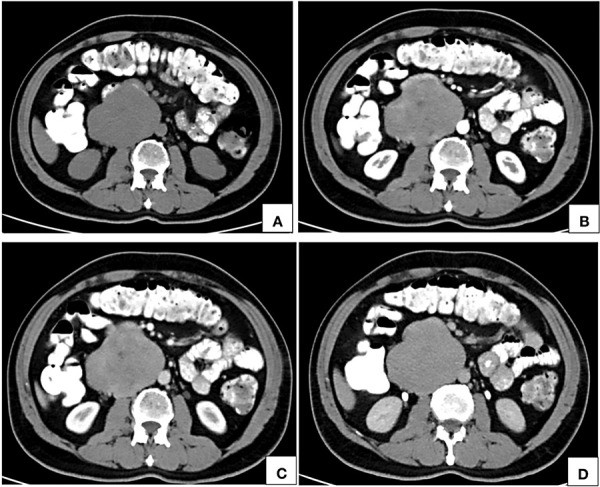
Computed tomographic axial images of noncontrast (A), arterial phase (B), portal venous phase (C) and delayed phase (D) show an isodense (A) retroperitoneal lesion right paramedian in location, in close contact with the abdominal aorta showing inhomogeneous areas of contrast uptake on arterial phase (B) with homogeneous enhancement on portal venous (C) and delayed phases (D). The lumen of inferior vena cava at the site of maximum contact is imperceptible.

**Figure 3.**
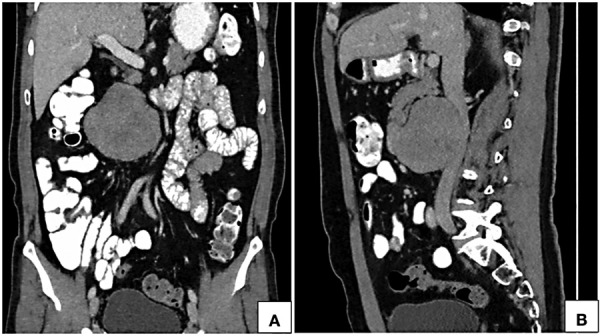
Computed tomographic coronal (A) and sagittal (B) reformatted images in portal venous phase show a near homogeneously enhancing retroperitoneal lesion in anterior relation to the infrarenal inferior vena cava towards the right of the midline. Proximal and distal segments of the inferior vena cava show normal contrast opacification with no intraluminal component.

Based on the radiological findings, differential diagnoses included low grade retroperitoneal lymphoma and primary retroperitoneal sarcoma. Histopathological sampling was done under computed tomographic guidance. Histopathological examination showed malignant proliferation of smooth muscle cells consistent with FNCLCC grade 2 leiomyosarcoma. The tumor was strongly positive for SMA and desmin, positive for vimentin and ß catenin and was negative for CK, CD 117, S 100, HMB 45 and LCA. Tumor cells showed 30% expression of Ki67. Immunihistochemical analysis was further confirmatory of leiomyosarcoma. Following histopathological examination and retrospectively evaluating the features on imaging, the possibility of primary leiomyosarcoma of infrarenal inferior vena cava was considered.

Patient then underwent laparotomy through midline approach for excision of the tumor. Intraoperatively, an exophytic circumscribed lobulated soft tissue tumor was seen arising from the anterior wall of infrarenal inferior vena cava. No intraluminal component was noted. Bilateral renal vessels were unremarkable. The tumor along with the corresponding segment of the inferior vena cava were excised and inferior vena cava graft reconstruction was performed. Bilateral renal veins were anastamosed to the reconstructed inferior vena cava. Excisional biopsy further confirmed the diagnosis.

**Figure 4.**
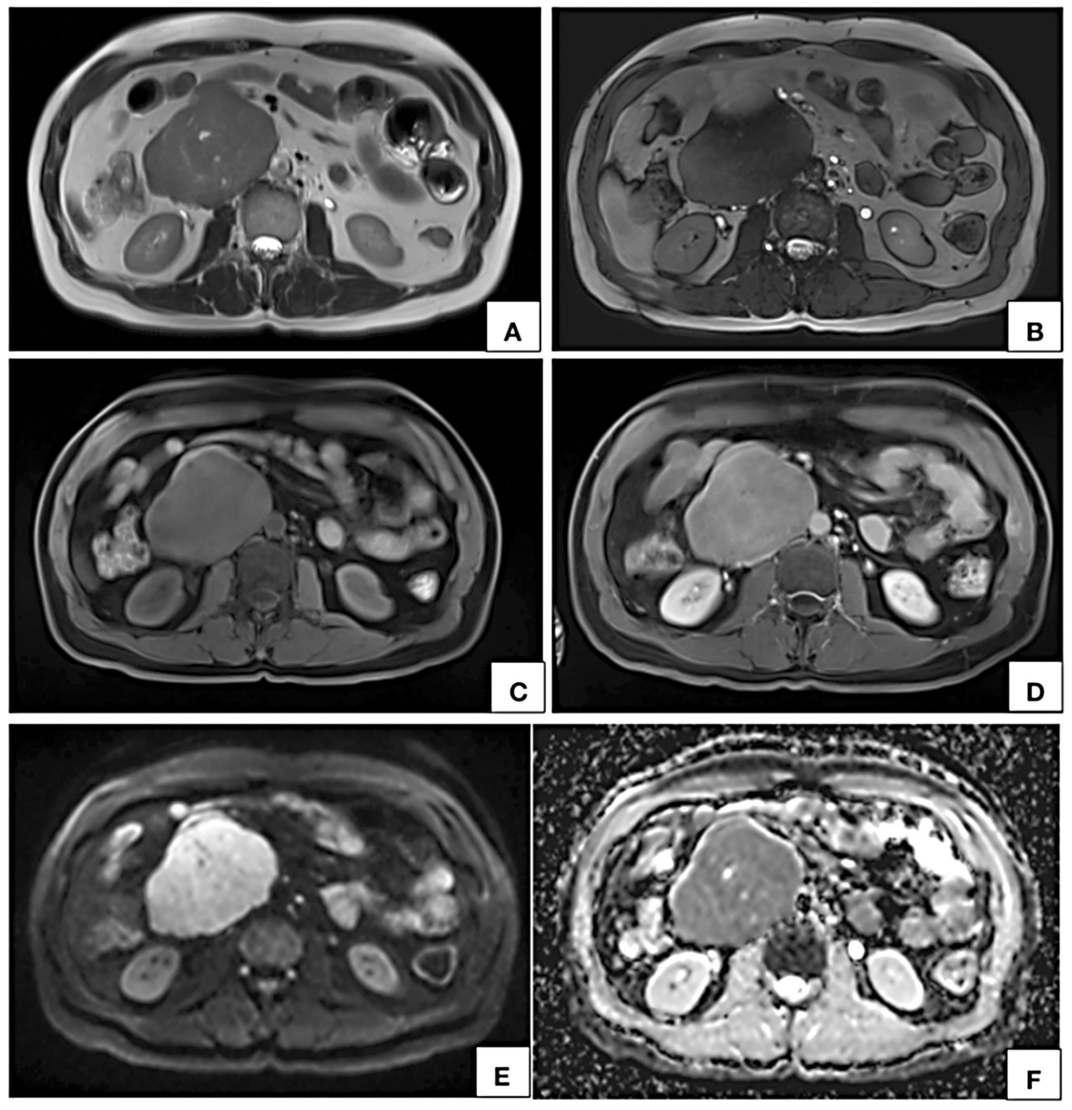
MRI axial T2W (A), T2W TRUFI (B), precontrast (C) and post contrast (D) T1W fat saturated VIBE images show a circumscribed lobulated retroperitoneal lesion on the right side of midline appearing hyperintense on T2W (A) and hypointense on T1W (C) showing near homogeneous contrast enhancement (D). In axial DWI (E) image and corresponding ADC map (F), the lesion shows restricted diffusion. Few T2W hyperintense nonenhancing cystic areas are seen within the lesion.

## Discussion

Leiomyosarcoma is the most common primary tumor of the inferior vena cava. Incidence is higher in females between 5^th^ and 6^th^ decade of life [5]. It is a slow growing plastic tumor that extends along the tissue planes of least resistance than directly invading the adjacent structures [7]. It generally presents as a retroperitoneal lesion and is often confused with primary retroperitoneal tumors. Surgical resection is the primary therapeutic option. Imaging plays a crucial role in preoperative evaluation of retroperitoneal tumors, in lesion characterization and assessment of spread. Differentiating LMS of inferior vena cava from other primary retroperitoneal tumors is a diagnostic challenge but carries high clinical implications in surgical planning. Preoperative differentiation of LMS of inferior vena cava (IVC) origin from a primary retroperitoneal tumor compressing the IVC is critical because primary origin from IVC will require optimal surgical planning for vascular repair or reconstruction. LMS of inferior vena cava can be classified on the basis of the involvement of IVC and on the basis of anatomical location of the tumor.

**Figure 5.**
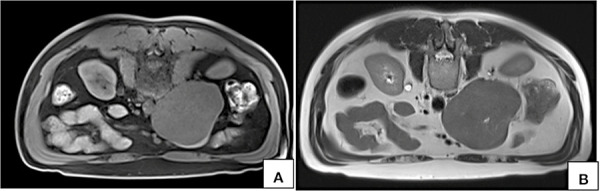
MRI axial T1W fat saturated VIBE (A) and T2W (B) images acquired in prone position show persistent nonvisualization of the lumen on inferior vena cava at the site of maximum contact.

**Figure 6.**
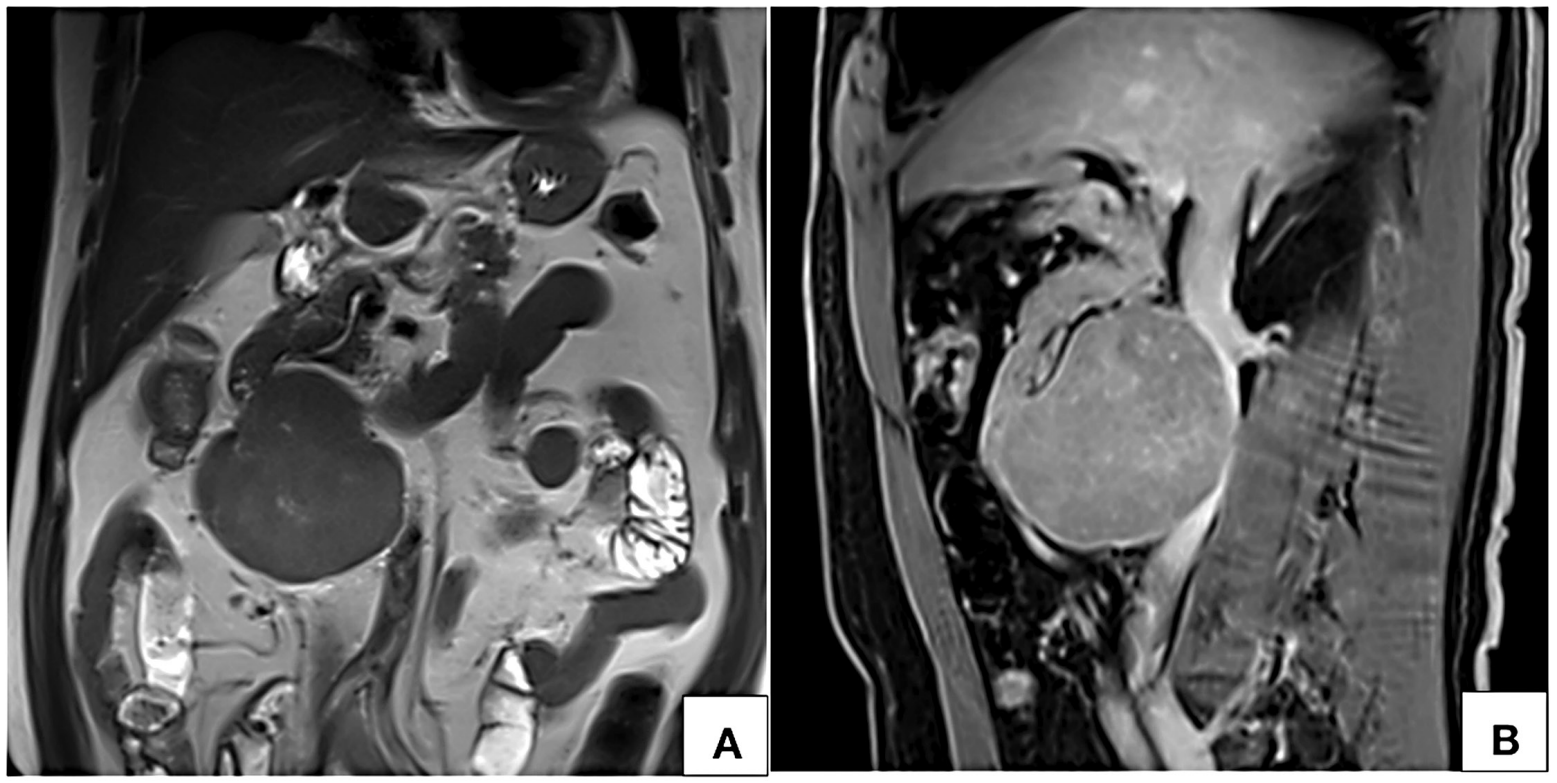
MRI coronal T2W (A) and sagittal post contrast T1W fat saturated VIBE (B) images show a circumscribed near homogeneously enhancing lesion along the anterior wall of infrarenal inferior vena cava with no intraluminal filling defect.

**Figure 7.**
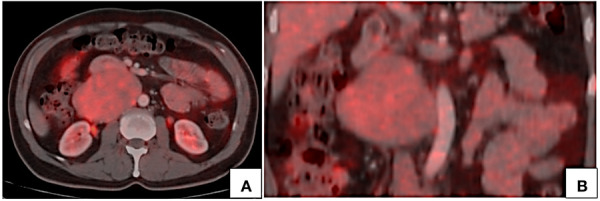
PET CT axial (A) and coronal (B) images show mild FDG uptake by the lesion with no hypermetabolic lymph nodes.

Classification based on IVC involvement [7]:

- Extraluminal – an exophytic mass lesion arising along the vascular mural wall of IVC.
- Intraluminal – soft tissue tumor within the lumen of IVC, extending along its course with high probability of extending into the right atrium.
- Mixed – shows both intraluminal and extraluminal components.

Classification based on anatomical location [8]:

- Segment I (Infra renal): tumor along the infrarenal part of IVC, commonly presents with pedal edema or back ache.
- Segment II (Inter renal or supra renal): tumor is seen at the origin of renal veins or above it without obvious involvement of the hepatic veins, presents with pain abdomen and renovascular hypertension.
- Segment III (Suprahepatic): involvement of hepatic veins with or without extension into the right atrium, presents as abdominal distension, hepatomegaly, vomiting, ascites and jaundice. Rarely presents with Budd–Chiari syndrome.

Common radiological features that aid in characterization of primary LMS of inferior vena cava in light of the existing literature are enumerated in table 1. In a large pooled analysis of 377 cases by Wachtel H et al (12), 133 (59.1%) cases were extraluminal, 56 (24.9%) were intraluminal and 36 (16%) cases had both intra- and extraluminal components. Similar results were reported by Mingoli et al (5), where out of a total of 144 cases, 106 (73.6%) cases had predominantly extraluminal tumor and 31 (21.5%) had intraluminal involvement. Tumors with intra- and extraluminal components are referred to as dumbbell tumors. Predominant intraluminal tumors and mixed tumors usually extend distally or proximally along the vascular lumen while extraluminal tumors generally show restricted or no extension as reported in our case. These tumors occur at a higher frequency in segment 2 of the IVC (i.e. between the origin of renal veins and confluence of hepatic veins) followed by segment 1 (infrarenal IVC) [5, 12, 14]. Tumors epicentered in the segment 2 had relatively higher incidence of extension into the hepatic veins and renal veins [3, 14, 17, 20]. Tumors originating from the suprahepatic segment (segment 3) of IVC commonly have a predominantly intraluminal component and show high prevalence of intracardiac extension [5, 13, 18]. Gafarli S et al reported an intraluminal tumor in segment 1 with transluminal extension into the right atrium, presenting with pulmonary thromboembolism [21]. Generally, intraluminal tumors from segment 1 show distal extension in to the iliac veins [5, 15]. Cortecero JM et al [13] reported an intraluminal leiomyosarcoma involving all three segments of the IVC extending into the right atrium and into the pelvis along right ovarian vein. Extraluminal LMS usually shows preserved interfaces with the adjoining organs. However, infiltration into the adjacent structures is not uncommon. Owing to the proximity of IVC to aorta, encasement of aorta is seen in aggressive tumors [5, 17, 19]. Extraluminal and mixed tumors with extraluminal component can also involve renal parenchyma, renal vascular pedicle and hepatic parenchyma (if occurring on right side) [9, 15, 17, 20]. Involvement of adrenal gland in a mixed tumor has also been reported earlier [14]. In 2004, Shvarts O et al [9] presented a mixed LMS of suprarenal (segment 2) IVC with extensive involvement of the right kidney that was initially misdiagnosed as a primary renal tumor. Li A et al [26] reported a case of large extraluminal LMS of segment 2 of the IVC that mimicked primary hepatocellular carcinoma with intravascular tumor thrombus. However in our case, fat planes with the adjacent structures were sharp with no obvious infiltration into surrounding structures.

**Table 1.** Salient radiological features for characterization and evaluation of leiomyosarcoma of the inferior vena cava from existing literature.

s. No	Study	No of cases	Type	Relation to IVC	Position	Relation with renal vessels	Extensions	MRI characteristics	Internal characteristics and enhancement	Other features
1	Mingoli et al, 1991 (5)	3 cases and a review of 141 cases								
	Case 1		Extraluminal	anterior to IVC, IVC compressed and displaced to the left	Segment 2	Uninvolved	NA	NR	NR	2cm inferior to pancreas
	Case 2		Mixed	posterior to IVC, in front of lumbar spine	Segment 1	Adherent	NR	NR	heterogeneous	Adherent to right kidney, ureter and iliac vessels, right common iliac vein thrombosis
	Case 3		Mixed	Left side of suprarenal IVC, displacing it to the right	Segment 2	Adherent	NR	NR	NR	Adherent to aorta and left renal vein
	Review of 141 cases		Predominantly extra-luminal- 105 (72.2%) Predominantly intraluminal- 29 (27.1%)	NA	Segment 1- 49 (34%) Segment 2-59 (41.7%) Segment 3-34 (24.3%)	NA	Hepatic veins-16 (11.1%) Right atrium- 32 (22.2%) Distal IVC-11 (7.6%)	NR	NR	Budd Chiari- 32 (22.2%)
2	Shvarts O et al, 2004 (9)	One	Mixed	Towards right	Segment 2	Right renal vein- involved	Segment 3 upto right atrium	NR	Heterogeneous	Right kidney, liver- involved, pulmonary metastases
3	Sessa B et al, 2010 (10)	Two								
	Case 1		Extraluminal	Anterior, towards right	Segment 2	Uninvolved	Absent	Hypointense on T1W, intermediate on T2W	Non homogeneous early enhancement with greater filling in venous phase	NR
	Case 2		Mixed	Anterior	Segment 1	NA	Absent	NR	Heterogeneous with necrotic areas	Infiltration of aorta
4	Chan G et al, 2014 (11)	One	Extraluminal (small tumor initially thought as caval lymph node, 2.7 cm)	Anterior, towards right	Segment 2	Uninvolved	Absent	NR	NR	Renal and pulmonary metastasis
5	Wachtel H et al, 2015 (12)	377	Extralu-minal-133 (59.1%) Intraluminal-56 (24.9%) Mixed- 36 (16%)	NR	Segment 1-78 (22.5%) Segment 2- 176 (50.7%) Segment 3- 19 (5.5%)	NR	Right atrium-19 (6.2%)	NR	NR	Budd Chiari- 3 (1.3%)
6	Cortecero JM et al, 2015(13)	One	Intraluminal	NA	Segment 1,2,3	Uninvolved	Right atrium, right ovarian vein	Intermediate on T1W, T2W	Mild, heterogeneous	NR
7	Teixeira FJR et al, 2017(14)	7	Extraluminal- 6 Mixed-1	Anterior- 6 Posterior- 1	Segment 1-2 cases Segment- 5 cases	Involved in 2 cases from segment 2	NR	NR	Heterogeneous	Pulmonary metastasis- 2 Cases Right adrenal involvement-1 case
8	Xu J et al, 2017 (8)	One (HIV positive)	Mixed (predominantly intraluminal)	NA	Segment 2	Bilateral renal vein thrombosis	Distally into segment 1	NR	Homogeneous	Bilateral iliac and femoral vein thrombosis.
9	Lopez Ruiz et al, 2017 (3)	One	Mixed	Anterior, towards right	Segment 2	Right renal vein- involved	Absent	NR	NR	NR
10	Sharma A et al, 2018 (15)	Three								
	Case 1		Extraluminal	Anterior, towards right	Segment 1	Uninvolved	Absent	NR	Heterogeneous	Adherent to duodenum and right ureter
	Case 2		Extraluminal	Anterior, towards left	Segment 1	Uninvolved	Absent	NR	Heterogeneous	NR
	Case 3		Mixed	Anterior, midline	Segment 1	Uninvolved	Bilateral common iliac veins	NR	Heterogeneous	Inferior to pancreas, close relation to 3^rd^ part of duodenum
11	Madhavan S et al, 2019 (16)	One	Extraluminal	Anterior, towards right	Segment 2	NA	Absent	NR	Homogeneous with calcifications	Extrinsic compression on CBD with resultant IHBRD
12	Lalwani AK et al, 2019(17)	One	Extraluminal	Anterior, towards right	Segment 2	Involved	Absent	NR	Heterogeneous	Involvement of right kidney and aorta
13	Zhou X et al, 2020 (18)	One	Predominantly intraluminal	NA	Segment 3	NA	Entrance of right atrium, right hepatic vein	Hypointense on T1W, hyperintense on T2W, with restricted diffusion	Heterogeneous with necrotic areas	Hepatic venous congestion in right lobe
14	Graves A et al, 2020 (19)	One	Extraluminal	Anterior, towards right	Segment 1	Uninvolved	Segment 2	NR	Heterogeneous	Partial aortic encasement
15	Rusu CB et al, 2020 (20)	One	Mixed	Right	Segment 2	Right renal vein- involved	Right renal fossa	NR	Heterogeneous	Infiltration of right kidney, right renal pedicle
16	Gafarli S et al, 2020 (21)	One	Intraluminal	NA	Segment 1	Uninvolved	Segment 2	NR	NR	Liver metastases
17	Pan J et al, 2021 (22)	20	Extraluminal- 9 (45%) Intraluminal- 1 (5%) Mixed-10 (50%)	NA	Segment 1-6 (30%) Segment 2-13 (65%) Segment3-1 (5%)	Total - 5 (25%) With renal involvement- 2 (10%) Without renal in-volvement-3 (15%)	NR	NR	NR	NR
18	Malki Y et al, 2021 (23)	One	Extraluminal	Anterior, towards right	Segment 1	Uninvolved	NR	NR	Heterogeneous with necrotic areas	NR
19	Lionberg A et al, 2021 (24)	One	Intraluminal	NA	Segment 2	NR	NR	NR	Heterogeneous	NR
20	Bilgo A et al, 2021 (25)	One	Extraluminal	Anterior, towards right	Segment 2	Right renal vein- involved	NR	NR	Heterogeneous with calcifications	NR
21	Li A et al, 2022 (26)	One	Extraluminal	Anterior, towards right	Segment 2	Uninvolved	NR	Hypointense on T1W, hyperintense on T2W	Heterogeneous with areas of necrosis and hemorrhage	NR

One of the important imaging feature for characterization of retroperitoneal tumors is the relationship with IVC. Webb Em et al [27] undertook a retrospective analysis of 13 cases of retroperitoneal tumors to study the performance of four different radiological signs in differentiating LMS of IVC from other primary retroperitoneal tumors. Radiological signs that were studied are positive embedded sign, negative embedded sign, imperceptible IVC sign and tumor in lumen. They observed that imperceptible IVC lumen at the site of maximum contact is highly predictive of a IVC origin. Case described in this manuscript had the imaging feature. Prone imaging can be of diagnostic utility in demonstrating primary origin from IVC in patients with predominantly extraluminal lesion. On prone imaging, these tumors show persistent contact with the wall of IVC or persistent nonvisualization of the lumen of IVC. Tumors with predominant extraluminal component commonly occur in anterior relation to the IVC and towards the right side of midline [9-11, 17, 19]. However, tumors originating posteriorly from the IVC have also been reported [5, 14].

These tumors commonly show heterogeneous enhancement with areas of necrosis within [9, 14, 15]. Sessa B et al [10] reported inhomogeneous early enhancement followed by homogenous filling in portal venous phase. We observed inhomogeneous contrast uptake in arterial phase followed by near homogeneous enhancement in portal venous and delayed phases with nonenhancing areas within. Few authors have also reported presence of calcification [16, 25] and hemorrhage [26] within the lesion.

On MR imaging LMS of IVC appears hypo- to intermediately intense on T1W and hyperintense on T2W [10, 18, 26]. Due to their malignant nature, these tumors also show diffusion restriction [18]. Similar signal characteristics were observed in our case also.

On Positron emission tomography (PET), these tumors show mild to intense uptake of fluorodeoxyglucose (FDG) [21, 28, 29]. Singh N et al [28] presented a case of intraluminal LMS showing intense FDG uptake with SUV_max_ value of 18. In the above presented case, the lesion showed mild FDG uptake with an SUV_max_ value of 4. PET CT has an indispensable role in the evaluation of intraluminal tumors in differentiating tumor thrombus from bland nontumor thrombus since these tumors present with venous thrombosis. Another significant role of PET CT lies in assessment of spread. These tumors can be locally aggressive but distant metastases are uncommon. However, lung is the most common site of metastasis [2, 11, 14]. Metastasis to the liver and kidney have also been reported [11, 21].

Important differential diagnoses include retroperitoneal sarcoma and lymphoma. Lymphomas show distinctive imaging features such as homogeneous enhancement, hypointense signal on T1W and T2W showing intense diffusion restriction and avid FDG uptake. Differentiating primary retroperitoneal sarcoma from leiomyosarcoma of the IVC is challenging. However, absence of perceptible lumen of IVC at the site of maximum contact and persistent contact with the wall of IVC on prone imaging are strong predictors of IVC leiomyosarcoma.

Histopathological sampling of the tumor is performed under ultrasonographic or computed tomographic guidance. In patients with intraluminal extension of the tumor, few authors have demonstrated conventional cavagraphy based transvenous sampling [8, 24].

En bloc surgical excision with negative surgical margins remains the main stay of treatment. Being tumors of vascular origin, these tumors require vascular repair in form of primary repair or graft based on the size and location of the tumor. Achieving clear surgical margin is the most significant factor affecting survival [30]. Patients with features of advanced disease undergo palliative chemotherapy and radiotherapy. Patients with clear resection margins are also treated with adjuvant chemotherapy and radiation therapy. However, the benefit of adjuvant chemoradiation is unclear [14].

## Conclusions

Leiomyosarcoma of the inferior vena cava is a rare tumor of vascular origin. Imaging plays an imperative role in the diagnosis and preoperative evaluation. These tumors commonly show heterogeneous enhancement and occur in the segment 2 of IVC at a higher frequency. Extraluminal form is common. PET CT plays a prime role in the diagnosis of intraluminal form of the tumor.

## Abbreviations:

IVC: Inferior vena cava
LMS: Leiomyosarcoma
CT: Computed tomography
MRI: Magnetic Resonance Imaging
PET: Positron Emission Tomography
FDG: Fluoro deoxy glucose
SUV: Standard Uptake Value
FNCLCC: French federation of Cancer Centres Sarcoma Group
TRUFI: True Fast Imaging with steady-state free precession
DWI: Diffusion Weighted Imaging
ADC: Apparent Diffusion Coefficient
VIBE: Volumetric Interpolated Breath-hold Examination
